# Pesticides in the population of European hedgehogs (*Erinaceus europaeus*) in Denmark

**DOI:** 10.3389/fvets.2024.1436965

**Published:** 2024-08-09

**Authors:** Sophie Lund Rasmussen, Peter Roslev, Jeppe Lund Nielsen, Cino Pertoldi, Katrin Vorkamp

**Affiliations:** ^1^Wildlife Conservation Research Unit, The Recanati-Kaplan Centre, Department of Biology, University of Oxford, Abingdon, United Kingdom; ^2^Department of Chemistry and Bioscience, Aalborg University, Aalborg, Denmark; ^3^Linacre College, University of Oxford, Oxford, United Kingdom; ^4^Aalborg Zoo, Aalborg, Denmark; ^5^Department of Environmental Science, Aarhus University, Roskilde, Denmark

**Keywords:** biocides, ecotoxicology, xenobiotics, environmental pollution, bioaccumulation, terrestrial environment, wildlife conservation

## Abstract

European hedgehogs (*Erinaceus europaeus*) inhabit most of Denmark, except for a few smaller islands. Research from other European countries has shown that the hedgehog populations are in decline. The exposure to chemicals might contribute to this development, although their role is currently unknown. Our research studied the occurrence of 19 selected pesticides in the Danish hedgehog population as well as factors potentially explaining the levels of chemicals detected. We analysed 115 liver samples obtained from dead hedgehogs in 2016 for seven rodenticides, four insecticides and eight herbicides commonly used in Denmark at the time of sampling, applying a high-performance liquid chromatography–tandem mass spectrometry (HPLC-MS/MS) method. Detection frequencies varied between 0.9% for fluroxypyr and trans-permethrin and 79% for bromadiolone. Rodenticides, insecticides and herbicides were detected in 84, 43, and 50% of the samples, respectively. The compounds most frequently detected included the insecticide imidacloprid (35%), the herbicide metamitron (29%) and the rodenticide bromadiolone (79%). Individual concentrations varied between non-detected to >2 μg/g. A total of 79% of the 115 hedgehogs contained more than one detectable pesticide, with up to nine of the 19 compounds detected in one individual. The detection frequencies were found to differ significantly between the Eastern and Western part of Denmark for difenacoum, difethialone and imidacloprid. However, no associations were found with sex, age, habitat type or the prevalence of *mecC*-MRSA and endoparasites in the hedgehogs tested. Whether or not the pesticide levels detected carry a health risk for the hedgehogs remains unknown as no adverse effect levels have yet been established for European hedgehogs for single compounds or pesticide mixtures.

## 1 Introduction

The European hedgehog (*Erinaceus europaeus*), hereafter referred to as “hedgehog,” is found throughout most of Denmark with the exception of a few smaller islands (1). The species is adaptable and can live in many different types of habitats, such as forests [especially deciduous forests, as well as in the transition zones between forest and open land (2)], agricultural and residential areas as well as public green spaces, such as city parks and cemeteries (3). However, research has shown that hedgehogs nowadays prefer to live in areas dominated by human settlement and activity, especially urban areas (4–8).

The hedgehog populations are declining in Europe, particularly in areas with cultivated land (9–13), and for some European countries, the hedgehog is listed as “vulnerable” (13), “threatened” (14) or “near threatened” (15–19) on the national Red Lists of the International Union of Conservation of Nature (IUCN). The decline in hedgehog populations can be attributed to habitat loss, landscape fragmentation, agricultural intensification, road kills, poisoning, domestic garden accidents, and in some areas also attacks by predators such as foxes, badgers and domestic dogs (4, 6, 7, 20–32). Due to the worrying decline documented in several European countries, it is important to study the potential underlying factors causing the population decline to inform hedgehog conservation initiatives.

Due to their ecology, hedgehogs can be exposed to pesticides and other potentially harmful chemicals which are used in their habitats, or which are omnipresent in the environment. Hedgehogs are officially classified as insectivores, but will also eat snails, slugs, earthworms, carrion, eggs and live vertebrates including amphibians and reptiles, if given the opportunity (33–37). In addition, many garden owners provide supplementary feeding with cat food, which is now thought to make up a significant part of the hedgehog diet in residential neighbourhoods (8, 38). Hedgehogs can be exposed to chemicals from their drinking water as well as from direct contact to soil or vegetation when travelling through the dense vegetation, including crops that may have been treated with herbicides or insecticides. They may eat rodenticide or insecticide products directly if they have access to them, and are otherwise potentially exposed to these via secondary poisoning from scavenging on poisoned mice or rats (26) or through the ingestion of poisoned invertebrates (39). In addition, hedgehogs are also exposed to ectoparasite insecticides via pets, as these are excreted from cats and dogs via their urine and faeces or may be washed off the skin and transferred to lakes or other sources of fresh water (40–42). Furthermore, hedgehogs that are admitted into care at wildlife rehabilitation centres are frequently treated with insecticides against fleas (43).

Our recent review showed that hedgehogs were exposed to various chemicals, including pesticides such as rodenticides, but also persistent organic pollutants (POPs), including polychlorinated biphenyls (PCBs), some organochlorine pesticides and brominated flame retardants (BFRs), and various metals and metalloids (44). The review was based on studies analysing different types of samples such as hair and spines, blood, adipose tissue, liver, kidneys and muscle (26, 45–57), and documented the co-occurrence of a range of hazardous chemicals in hedgehogs, with potential implications for hedgehog health and population development (44). The aim of the current study was therefore to investigate the presence of different types of pesticides in Danish hedgehogs, to discuss potential factors determining hedgehog exposure and to expand the general knowledge base of the occurrence of these chemicals in terrestrial wildlife.

## 2 Materials and methods

### 2.1 Hedgehog samples

A total of 697 dead hedgehogs were collected in Denmark in 2016 during a citizen science project, The Danish Hedgehog Project (58), with the majority of hedgehogs having died in collision with cars. Of these 697 individuals, 411 contained intact livers for further analyses, which were removed during necropsies and stored at −20°C until the chemical analysis was conducted. Liver samples of 115 individuals were selected for the chemical analysis of pesticides (rodenticides, herbicides and insecticides). The samples were selected to cover a broad age range (59), a wide geographical representation of Denmark (including urban and rural habitats) and both sexes. Furthermore, samples were prioritised if information was available on date and cause of death (traffic, in care, natural), and the prevalence of endoparasites (60) and methicillin-resistant *Staphylococcus aureus* (MRSA) (61). The cause of death described as ‘natural’ refers to hedgehogs found dead in the wild from causes other than collisions with cars.

### 2.2 Selection of pesticides for the study

As the dead hedgehogs used in the study were collected in 2016, we used official records of the use of pesticides in Denmark in 2016, 2017 and 2019 to select the most relevant and commonly used pesticides for the analysis (62–65). We selected seven rodenticides (bromadiolone, coumatetralyl, brodifacoum, difenacoum, difethialone, chloralose (consisting of the isomers α-chloralose and β-chloralose)), four insecticides [imidacloprid, permethrin (consisting of *cis*- and *trans*-permethrin isomers), and fipronil] and eight herbicides [metamitron, 2-methyl-4-chlorophenoxyacetic acid (MCPA), 2,4-dichlorophenoxyacetic acid (2,4-D), diflufenican, prosulfocarb, bentazon, pendimethalin and fluroxypyr], which could be combined in the same analytical method. Details of the selected compounds, as well as the selection process, are provided in the Supplementary materials.

### 2.3 Chemical analysis of the selected pesticides

**Figure 1:**
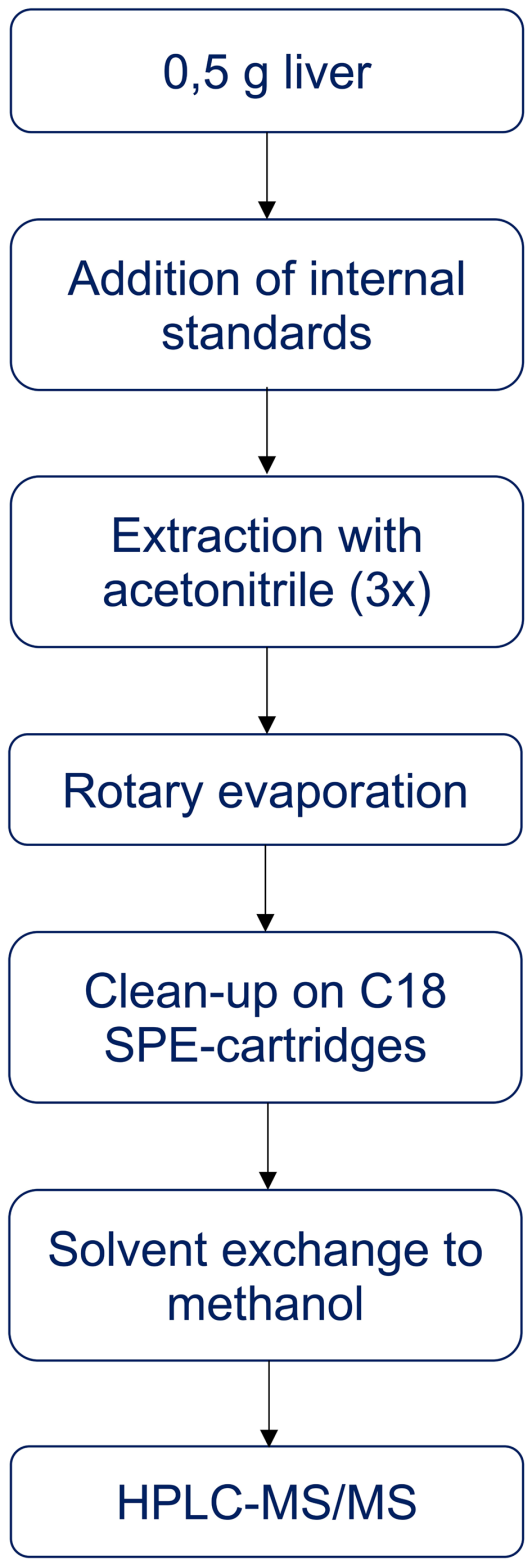
Summary of sample preparation for the analysis of selected substances.

The analytical method was based on Albert et al. (66), with modifications, and is summarised in Figure 1. For the analysis, 0.5 g of liver was weighed and mixed with anhydrous Na_2_SO_4_ to dry the sample. After addition of recovery standards (tebuconazole-d6, 97% purity, Dr. Ehrenstorfer, Germany, and propiconazole-d5, >94% purity, Sigma Aldrich, USA) and internal standards (^13^C-MCPA,^13^C-2,4-D, ^13^C-imidacloprid, all 99% purity from Cambridge Isotope Laboratories, USA, bromadiolone-d5 and brodifacoum-d4, both ~99% purity, Toronto Research Chemicals, Canada), the samples were extracted with 7 mL acetonitrile (LiChrosolv^®^, Supelco, Merck, Germany) in an ultrasonic bath (10 min), followed by 2 min on a whirl mixer and 10 min centrifugation (3000 rpm). The solvent phase was transferred to a new flask and the extraction was repeated twice with 5 mL acetonitrile. The extract was evaporated to 1 mL using a rotary evaporator.

In the next step, the extract was purified on a solid phase extraction (SPE) C18 column (Sep-Pak, tC18 Vac Cartridge, 1 g, 6 cc, Waters, USA), with Na_2_SO_4_ on top to retain any water in the extracts. The columns were conditioned with 10 mL of acetonitrile. After elution with 8 mL acetonitrile, the sample was evaporated to dryness on a rotary evaporator and under N_2_, and the compounds were redissolved in 1 mL methanol (LiChrosolv^®^, Supelco, Merck, Germany). The final extract was filtered (0.2 μm) into a vial for analysis by high performance liquid chromatography and tandem mass spectrometry (HPLC-MS/MS; Agilent) using an InfinityLab LC column (Poroshell 120 EC-C18). Eluent A was MilliQ water with 5 mM ammonium acetate, and eluent B was methanol with 5 mM ammonium acetate. The analysis was performed with an A:B solvent gradient starting at 95:5, changing to 50:50 and ending at 0:100. For each substance, three ions were collected.

The 115 samples were analysed in six batches. Each batch also included a blank and two calf liver samples that had been spiked with all substances and therefore acted as positive controls. The recovery in the spiked control samples was used to correct the results from the same batch if the recovery was <80%. The substances that had a matching internal standard (MCPA, 2,4-D, imidacloprid and bromadiolone) were quantified using this labelled standard, while the other substances were quantified with an external calibration. The calibration consisted of two sets of standards with 12 concentration levels.

The method detection limits (MDLs) were determined as the lowest standard with a signal/noise ratio of 3, normalised to the individual sample intake. A list of MDLs is provided in the Supplementary Table 1. MDLs generally ranged between 0.05 and 0.39 ng/g wet weight but increased by a factor of about 2.5 for three samples for which only approximately 0.2 g hedgehog liver were available for analysis.

### 2.4 Statistical analyses

#### 2.4.1 Categorisation of samples for statistical analysis

**Figure 2:**
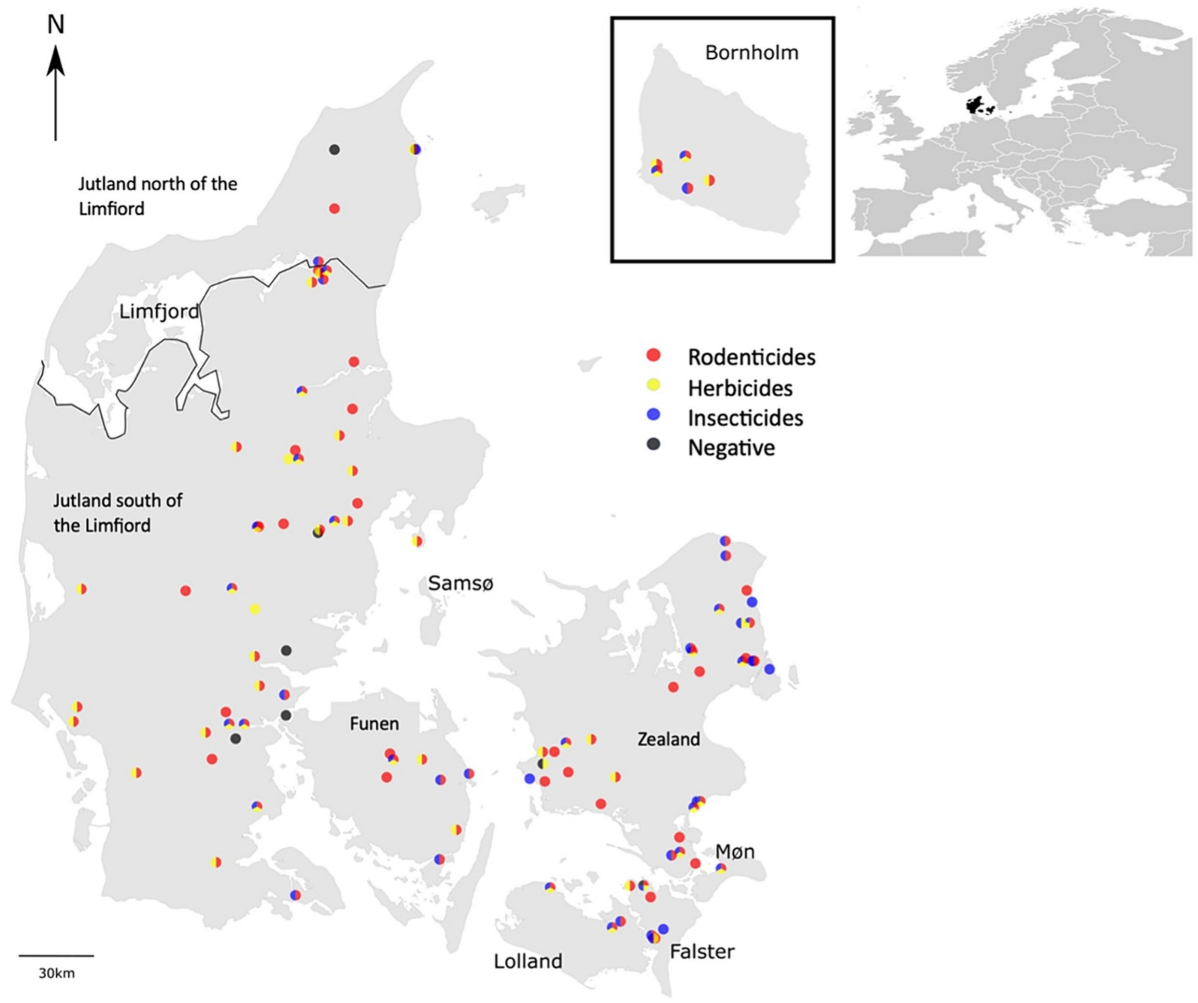
Geographical distribution of the 115 hedgehog samples included in the study and the categories of pesticides found in the individual individuals.

The 115 hedgehogs selected for analysis included 43 females, 69 males and 3 individuals of unknown sex. The individuals were grouped into eight different geographical regions, depending on their origin in Denmark (Figure 2): Zealand (*N* = 37), Jutland north of the Limfjord (*N* = 5), Møn (*N* = 1), Lolland (*N* = 3), Funen (*N* = 8), Falster (*N* = 9), Bornholm (*N* = 8), and Jutland south of the Limfjord (*N* = 47).

The hedgehogs were also divided into age groups (11 age classes in total), ranging from 0 to 16 years (59). However, only one or two individuals were represented in the age groups above 6 years: 0 years (*N* = 34), 1 year (*N* = 16), 2 years (*N* = 11), 3 years (*N* = 16), 4 years (*N* = 14), 5 years (*N* = 12), 6 years (*N* = 5), 9 years (*N* = 2), 10 years (*N* = 2), 11 years (*N* = 1), 13 years (*N* = 1), 16 years (*N* = 1) (see Supplementary Table 2).

The hedgehogs were also categorised by habitat types as described in Rasmussen et al. (61): urban (*N* = 62) and rural (*N* = 50). Three individuals lacked information on location and were therefore excluded from the test for the effect of habitat type on the prevalence of pesticides in hedgehogs. In addition, groups of hedgehogs carrying endoparasites (60) or MRSA (61) were tested against groups which tested negative for these parameters, to investigate potential pesticide-induced immunotoxicity. Finally, potentially lactating females (from 1 year of age) and non-lactating females (less than 1 year old) were compared.

#### 2.4.2 Statistical tests

For each compound included in the analysis, the following parameters were calculated: detection frequency (DF) and mean values. The insecticide fipronil was excluded from any further calculations as it was below the MDL in all samples.

Shapiro–Wilk tests were applied for the values of pesticide concentrations, and significant deviations from normality (*P* < 0.05) were found, due to skewed distributions. For this reason, Mann–Whitney *U-*tests (67) were performed to test whether the median values of pesticide concentrations varied between the groups of hedgehogs according to sex, regions, habitat types (rural or urban), in hedgehogs carrying endoparasites or not, in hedgehogs carrying *mecC*-MRSA or not, and between potentially lactating females (from 1 year of age) and non-lactating females (less than 1 year old). The median values were estimated for all the individuals; the individuals having pesticide concentration below the MDLs were considered with a concentration equal to 0. Fisher’s Exact test was used to investigate whether there was a difference in the detection frequencies between regions. Results with a detection frequency < 5% were excluded from the statistical tests, as described in Campbell (68).

Pearson’s product–moment correlation test was used to investigate whether the pesticide concentrations were correlated with the age of the individuals. The tests were performed on all individuals at once (with males and females pooled), as well as on males and females separately. If there were < 3 data points, the test was not performed.

To test whether there were significant differences in the median concentrations in the individuals with different causes of death (death in care, road fatality or natural death in the wild) we used the Kruskal-Wallis test. We then also tested this effect for the substances that showed significance in the Kruskal-Wallis test (bromadiolone and metamitron), by performing pairwise Mann–Whitney *U*-tests, where we tested the groups against each other pairwise.

For all tests, the different individuals were pooled together for all the factors we tested, to achieve adequate statistical power. As an example, when testing for differences between regions, results for individuals of both sexes, as well as all age groups and causes of death were used in the sample pool. Due to the large number of tests, the data was corrected with an overall Bonferroni correction (69), and based on the approach described in Miller (70) we created separate probability statements for each of the chemical substances included in the statistical analyses.

## 3 Results

**Figure 3:**
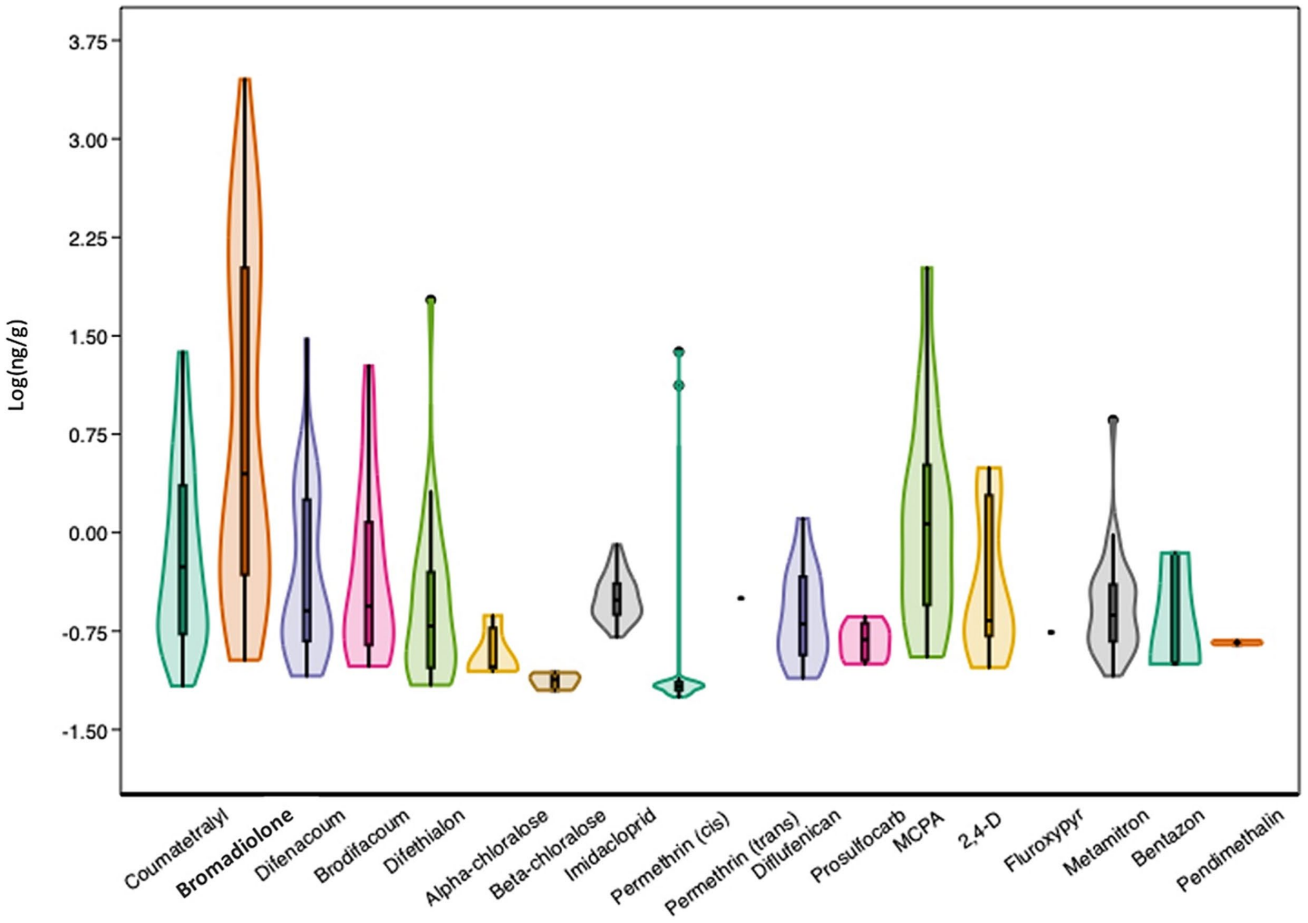
Violin plot with outliers and a presentation of the first (wide line, 25%) and third (narrow line, 75%) quartiles of the log-transformed concentrations (log ng/g) for the 19 tested substances in the livers of 115 hedgehogs.

With the exception of fipronil, the 19 substances selected for chemical analyses were widely detected in the samples, with varying detection frequencies across the samples and different patterns within the sample. Table 1 and Figure 3 provide an overview of the results for the 115 hedgehog liver samples (further details are provided in the Supplementary Table 2). As shown in Table 1, the concentrations vary widely, both between compounds and between individuals. Bromadiolone was the compound with the highest detection frequency as well as the highest mean and maximum concentration, which was close to 3 μg/g. In two other individuals, a concentration above 1 μg/g was found, while two other samples contained close to 1 μg/g, indicating that the high exposure situation was not a unique case. Furthermore, bromadiolone was the pesticide with the highest concentration in 71 (62%) of the samples. Thus, bromadiolone concentrations spanned five orders of magnitude between lowest (undetectable) and highest concentrations in the hedgehog livers.

**Table 1:** Analysis of 19 selected pesticides in 115 hedgehog liver samples.

	Rodenticides							Insecticides			Herbicides							
	Couma-tetralyl	Broma-diolone	Difena-coum	Brodi-facoum	Difethi-alon	*α*-chloralose	*β*-chloralose	Imida-cloprid	Perme-thrin (*cis*)	Perme-thrin (*trans*)	Diflu-fenican	Prosul-focarb	MCPA	2,4-D	Fluro-xypyr	Meta-mitron	bentazon	Pendi-methalin
*N*	45 (39.1%)	91 (79.1%)	31 (27.0%)	37 (32.2%)	31 (27.0%)	4 (3.5%)	3 (2.6%)	40 (34.8%)	22 (19.1%)	1 (0.9%)	13 (11.3%)	5 (4.3%)	14 (12.2%)	14 (12.2%)	1 (0.9%)	33 (28.7%)	3 (2.6%)	2 (1.7%)
Min (ng/g)	0.07	0.11	0.08	0.10	0.07	0.09	0.06	0.16	0.06	0.32	0.08	0.10	0.11	0.09	0.17	0.08	0.10	0.14
Max (ng/g)	23.68	2832.77	29.72	18.58	59.22	0.23	0.09	0.81	23.83	0.32	1.28	0.23	103.64	3.11	0.17	7.20	0.70	0.15
Mean (ng/g)	0.95	118.28	0.5	0.58	0.62	0.004	0.002	0.12	0.33	0.003	0.04	0.01	1.19	0.10	0.002	0.15	0.01	0.003

Information presented includes detection frequencies in terms of number (*N*) and percentage (%), minimum concentrations above the method detection limit (Min; ng/g), maximum (Max; ng/g) and mean values (Mean; ng/g) of the pesticide concentrations. The insecticide fipronil is omitted from the table as it was not detected above the method detection limit.

The second highest maximum concentration (for MCPA) was more than 20× lower than the highest bromadiolone concentration and was not found in the same individual. Instead, MCPA was the only compound above MDL in that specific liver sample. Fipronil was the only substance, which was not detected in any of the samples. Coumatetralyl, difenacoum and *cis*-permethrin also had relatively high maximum concentrations between 20 and 30 ng/g, despite relatively low overall detection frequencies (<40%; Table 1). Interestingly, the individuals with the highest concentrations of coumatetralyl and difenacoum also had elevated levels of bromadiolone (189 and 163 ng/g, respectively). Each individual also had elevated concentrations of other pesticides (MCPA and brodifacoum, respectively), but with no typical pattern. In the individual with elevated levels of *cis*-permethrin, nearly all other compounds were below MDL (See Supplementary Table 5).

No individual contained detectable levels of all the 19 pesticides included in the study at once. The number of compounds above MDL in an individual ranged from 0 (in eight samples, i.e., 7% of all samples) to 9, with an average of three compounds above MDL. A total of 79% (*N* = 91) of the 115 hedgehogs contained more than one detectable pesticide, but with no clear pattern (see Supplementary Table 3 for an overview of number of detected pesticides per individual).

**Figure 4:**
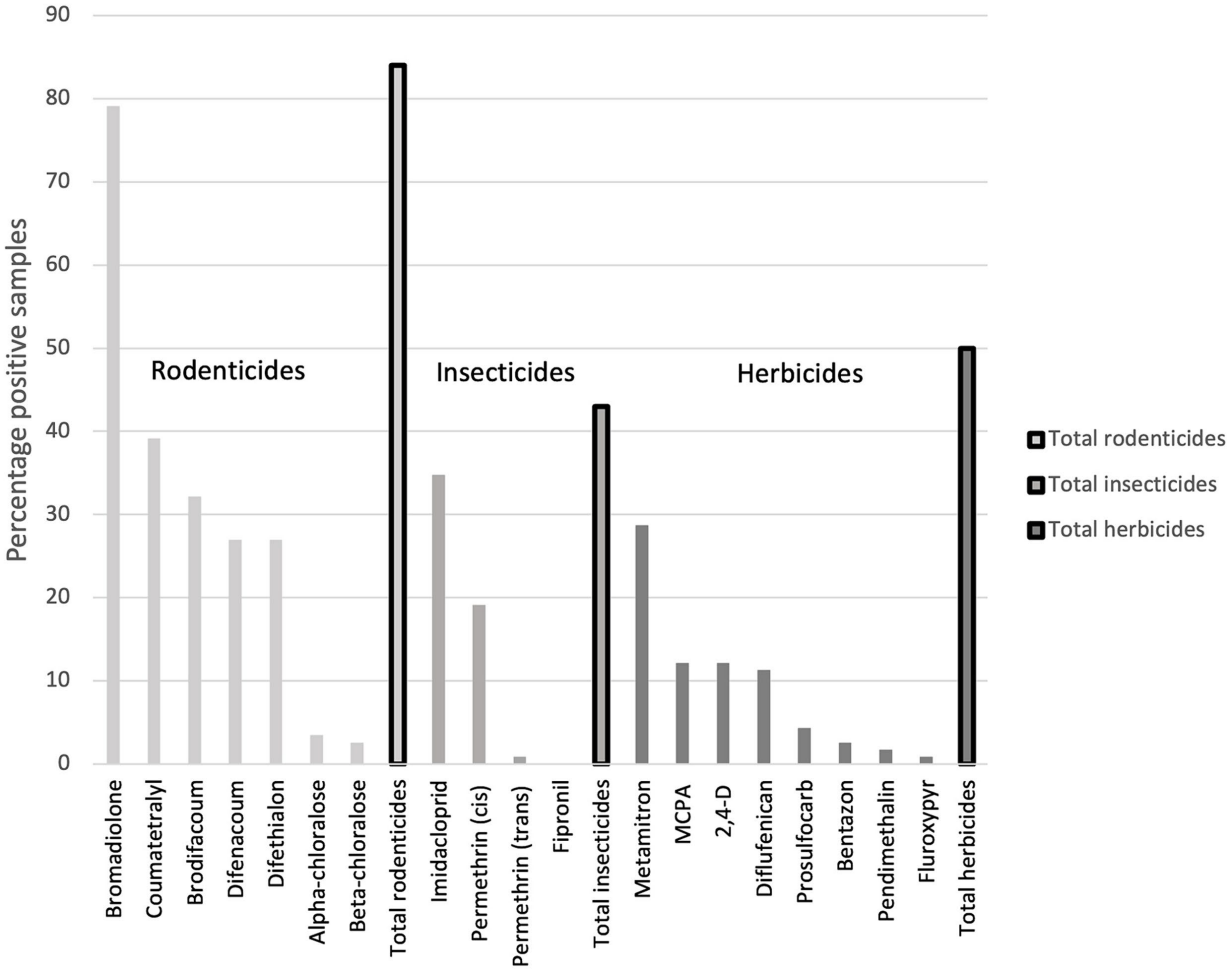
Detection frequency (%) of each of the 19 chemical substances analysed in Danish hedgehogs (*N* = 115), divided into rodenticides, insecticides and herbicides.

Rodenticides, insecticides, and herbicides were found in 84, 43, and 50% of the samples, respectively (Figure 4). Amongst the insecticides, the first-generation nicotinoid imidacloprid had the highest detection rate of 34% and a maximum concentration of 0.82 ng/g, whereas permethrin was only detected sporadically, although with a maximum concentration of 24 ng/g. Although MCPA was the herbicide with the highest maximum and mean concentration, it had a lower detection frequency than one other herbicide, metamitron, which had the highest detection rate (29%), also showing a relatively high maximum concentration of 7.2 ng/g (Table 1).

Detection frequencies and median and minimum and maximum values for the eight categorised regions are shown in the Supplementary Table 4. The median values shown in the Supplementary materials have been estimated only for the individuals which had pesticide concentration above the MDLs, and the minimum values refer to the lowest values found above the MDLs. We found no effect on the median values (estimated including all individuals; those having pesticide concentrations below and above the MDLs) of the concentrations of the pesticides selected for our chemical analyses for any of the factors: sex, habitat type, presence of endoparasites or MRSA, status of lactation, even before the Bonferroni correction was performed (*P* > 0.05).

Likewise, the correlation test showed no effect of age on the concentration of chemical substances (*R*-values: −0.17–0.115; *P*-values: 0.07–0.99), neither for sex, with males separately (*R*-values: −0.24–0.20; *P*-values: 0.051–0.98) and females separately (*R*-values: −0.27–0.25; *P*-values: 0.08–0.53). This was the case even before the Bonferroni correction was performed.

The median values of the concentrations of the chemical substances for each of the grouped geographical regions were tested with Kruskal Wallis tests followed by pairwise Mann–Whitney *U-*tests, and both tests showed no significant difference between the regions (*P* > 0.05).

However, when we tested for the effect of the cause of death on the concentrations of the substances, we found that road-killed hedgehogs had higher concentrations of bromadiolone in their bodies compared to hedgehogs that died of natural causes (*P* = 0.04). Furthermore, when testing for the effect of geographical location on the prevalence of pesticides in the hedgehogs, significant differences were found between different regions of Denmark (Figure 2). The rodenticide difenacoum had a higher prevalence in hedgehogs from Jutland south of the Limfjord (46.5%) compared to Zealand (21.6%) (*P* = 0.03). The opposite was the case for the rodenticide difethialone and the insecticide imidacloprid, which both had a higher prevalence in individuals from Zealand (37.8 and 45.9% for difethialone and imidacloprid, respectively) compared to individuals from Jutland south of the Limfjord (16.3 and 23.2%, respectively) (*P* = 0.04).

## 4 Discussion

### 4.1 Rodenticides

We detected at least one of the three types of pesticides we investigated, i.e., rodenticides, insecticides and herbicides, in 84, 43, and 50% of the samples, respectively. The rodenticide bromadiolone was the compound with the highest detection frequency, maximum and mean concentration. Some of our results were similar to those of a study on hedgehogs from the UK (26), however, with distinct differences in concentration levels. The British study from 2010 investigated the presence of six different rodenticides in hedgehogs that had died in care in England from 2004 to 2006 and found that a total of 67% of 120 hedgehogs contained rodenticides in their livers. The British study included four of the same rodenticides analysed in our study, i.e., bromadiolone, difenacoum, coumatetralyl and brodifacoum. However, coumatetralyl was not investigated in hedgehogs by Dowding et al. (26). Interestingly, difenacoum was the most frequently detected rodenticide in the UK study, i.e., found in 48% of samples (*N* = 57/120) (26). In the current study, we found difenacoum in 27% of the samples (Table 1), whereas bromadiolone was found in 79% of all samples, compared to 19% in the UK samples. However, these detection frequencies are not necessarily directly comparable because of differences in the analytical methods, including the method detection limits. In fact, the British samples showed higher mean concentrations than the Danish for all three substances. Bromadiolone (DK = 118 ng/g versus UK = 590 ng/g), difenacoum (DK = 0.5 ng/g versus UK = 100 ng/g), and brodifacoum (DK = 0.6 ng/g versus UK = 50 ng/g).

A study screening hedgehogs dying in care (*n* = 48) in Spain from 2011 to 2013 for six anticoagulant rodenticides in liver samples (57), found a frequency ranging from 0% (warfarin) to 50% (brodifacoum) and detected anticoagulant rodenticides in 28 out of the 48 individuals, with a total mean concentration of 122 ng/g anticoagulant rodenticides per individual (57). Despite lower detection frequencies, compared to our study, difenacoum, brodifacoum and difethialone all had much higher mean concentrations (57) than those found in our study (Table 1). One exception was the compound bromadiolone, where a mean concentration of 78.8 ng/g was calculated in the Spanish study compared to 118 ng/g in the present study. In another study on livers from two hedgehogs dying of suspected poisoning at a wildlife rehabilitation centre in Spain in 2005–2010, the analysis of six different rodenticides revealed bromadiolone (*N* = 2/2, mean 26 ng/g, range 13–49 ng/g) and brodifacoum (*N* = 1/2, mean 92 ng/g), while others, including coumatetralyl, could not be detected (54).

A Danish study from 2015 (71) described a high prevalence of bromadiolone in samples of stone martens *(Martes foina)* (95–100%) and polecats *(Mustela putorius)* (90–97%), and a lower prevalence of between 5 and 21% in small mammals such as mice and shrews. The high detection frequency in carnivores is interesting, as hedgehogs are scavengers and are thus also susceptible to secondary poisoning by eating poisoned mice and rats. The study found concentrations of bromadiolone between 0 and 364 ng/g in small mammals, and up to 2355 ng/g for stone martens (71). We detected concentrations of bromadiolone between <MDL-2833 ng/g in the hedgehogs, which is on par with the concentrations found in the stone martens, which also prey on small mammals. These findings, together with our detection of rodenticides in 84% of the analysed hedgehogs, and bromadiolone in 79% of the samples, indicate that rodenticides, especially bromadiolone, are widely present in terrestrial animals in Denmark. However, it is noteworthy that most rodenticide concentrations have a range of several orders of magnitude in the liver samples analysed in this study, indicating a substantial variation in exposure. Assessments of potential health impacts on hedgehogs should therefore not only be based on median or mean exposure levels, but also consider these high exposure situations.

### 4.2 Insecticides, herbicides and other pesticides

Our study showed a wide presence of insecticides and herbicides in hedgehogs, but slightly lower detection frequencies compared to the occurrence of rodenticides. Other types of pesticides were not included in the study. Previous research into the prevalence of insecticides and herbicides in hedgehogs has been limited, and often applying relatively small sample sizes (44). In a study screening for 55 pesticides (insecticides, fungicides, herbicides, and nematicides) in livers from six hedgehogs, dying at a wildlife rehabilitation centre in Germany (55), only six compounds were detected. These included the fungicides fenpropimorph and tebuconazole, the insecticides dieldrin and permethrin, as well as the metabolites fipronil sulfone (originating from the insecticide fipronil) and p,p’-DDE (originating from the insecticide p,p’-DDT) (55). The detection frequencies for insecticides ranged between 17 and 50%, apart from a 100% representation of fipronil (as fipronil sulfone), which could be explained by routine treatment with fipronil of all admitted hedgehogs to the rescue centre (55). In the present study, we found between 0 and 35% positive samples for the four selected insecticides, with a total detection of insecticides in 43% of the samples. Fipronil was not detected in any of the samples of our study. However, the degradation product fipronil sulfone was not included in the study and we argue that fipronil, if present, may have been degraded to fipronil sulfone and other metabolites (58). Hence, fipronil metabolites would be obvious candidates for future studies. Neither did our study include any of the insecticides classified as POPs under the Stockholm Convention of the United Nations and banned in Denmark long before our samples were collected, such as dieldrin and p,p’-DDT/p,p’-DDE. Permethrin was detected in one of the individuals of the German study, at a relatively high concentration of about 7 ng/g, clearly exceeding the mean, but not the maximum concentration of our study (Table 1).

Further studies have described few or single cases of suspected poisoning in hedgehogs, with carbamate insecticides detected in the hedgehogs (72–74). Research into the effects of metaldehyde on hedgehogs has revealed that hedgehog may eat up to 200 poisoned slugs without showing signs of (secondary) poisoning (75). In addition, Keymer et al. (76) found concentrations of up to 80 μg/g of acetaldehyde (a by-product and metabolite of metaldehyde) in three hedgehogs dying from suspected metaldehyde poisoning in the UK between 1976 and 1986, showing that lethal threshold levels for these types of insecticides are relatively high. The insecticide concentrations in our study were generally much lower, however, toxic concentrations cannot be directly compared between insecticides.

Neonicotinoids, such as imidacloprid, are the largest group of commercial insecticides, mainly used for seed coating (77). They target nicotinic acetylcholine receptors in insects (78), however, effects observed on aquatic insects and honeybees in particular have raised doubts about their safe use (79, 80). Imidacloprid can persist in soil, and effects on soil amoeba were shown recently (81). Since imidacloprid and two other neonicotinoids are now banned on field crops in the European Union, exposure to hedgehogs will likely decrease. However, subsequent generations of neonicotinoids are still on the market, with unknown exposure to, or effects on, hedgehogs. Amongst the herbicides, the 1,2,4-triazine metamitron had the highest detection frequency (29%), while the phenoxy acid MCPA, which can also be a transformation product, had the highest maximum and mean concentration (Table 1). The results indicate that neither of the compounds are applied in a widespread or uniform way. However, their use can lead to relatively high exposure cases in hedgehogs, exceeding the maximum insecticide levels by 1–2 orders of magnitude. Whether these relatively high levels reflect recent exposure or accumulation over time, is unknown from our data. Both compounds have half-lives of about 10–60 days in soil, but lower half-lives have been observed in some field trials under real environmental conditions (82, 83). Given its high water solubility and low sorption to soil, MCPA is mainly associated with residues in water bodies.

### 4.3 Factors determining the occurrence of pesticides in Danish hedgehogs

Although the results cover a large variation in pesticide concentrations, there is little evidence of general region-specific patterns. However, we found three statistically significant differences in the detection frequencies between regions. For the rodenticide difenacoum, there was a higher prevalence in hedgehogs from Jutland south of the Limfjord (46.5%) compared to Zealand (21.6%), while the opposite was the case for the rodenticide difethialone and the insecticide imidacloprid. This could be indicative of different patterns of use between different regions, but the reasons remain speculative. Imidacloprid is used in pet flea control products, so its occurrence could be related to the number of pets, in particular dogs and cats, in different regions. However, there is no major difference in these numbers for the two parts of the country (84).

The statistical analyses showed no evidence of differences in the occurrence of pesticides in male and female hedgehogs. Previous research has shown that females of other animal species can reduce their body burden of POPs through lactation, as the substances are transferred via the mother’s milk to the offspring (85, 86). This is mainly related to lipophilic POPs that accumulate in the high lipid content of the milk. However, the fat content of hedgehog milk is only 10%, in contrast to, e.g., 28–26% for polar bears (*Ursus maritimus*) (87, 88), which may explain why we failed to detect a difference between the levels of pesticides in potentially lactating vs. non-lactating hedgehogs. Accumulated POPs are transferred from the fat to the blood stream and can reach the liver (89, 90), when the fat is metabolised, especially in periods of stress, where an animal is starving. However, since both sexes rely equally on substantial fat reserves to survive hibernation, we do not expect sex differences in contaminant mobilisation in European hedgehogs. Furthermore, the physico-chemical properties of the substances of this study are different from those of typical POPs, as they are less lipophilic and easier to degrade. For this reason, processes known from research on POPs will likely not apply to the same extent to less persistent pesticides.

The statistical analysis indicated that bromadiolone concentrations were significantly higher in road-killed hedgehogs compared to individuals found dead from other causes in the wild. However, the association might not be a causal one and other explanations for this result could be the higher representation of road-killed hedgehogs (*N* = 43) in the sample size of individuals with concentrations of bromadiolone above the detection limit, compared to rehabilitated individuals (*N* = 20) and individuals dying in the wild from other causes than car collisions (*N* = 28), and a varied representation of age groups across the three categories of cause of death. The fact that bromadiolone was the only compound that showed any association, might also be related to its high detection rate of 79%, resulting in more values different from zero.

In general, the low detection frequencies of most of the compounds resulted in small sample sizes and affected the statistical analyses. For the pairwise comparisons through the Fisher’s Exact test on the detection of specific pesticides per geographical region, or detection of specific pesticides per cause of death, the sample sizes were so small that the results may not have been significant, had the Bonferroni correction been applied.

### 4.4 Health implications

To date, only a few studies have documented health effects in hedgehogs caused by exposure to xenobiotics, such as a few fatal cases of poisoning with high concentrations of rodenticides (54, 91–93). In a study from Spain the wildlife carcasses chosen for rodenticide screening were suspected to have died from poisoning by anticoagulant rodenticides due to discernable hemorrhages detected during necropsies (68). A total of 32 hedgehogs targeted with rodenticides as part of the pest eradication programme in New Zealand were found dead and later confirmed as having been poisoned (92). Furthermore, high hepatic metal(loid) concentrations were associated with biliary hyperplasia in hedgehogs, suggesting that heavy metals and metalloids may be the primary contributing factor causing biliary hyperplasia in hedgehogs (56).

Histological examination of organs from the hedgehogs included in our study could possibly provide indications on potential health effects of the presence of the pesticides we detected in the hedgehogs. However, as discussed in Rasmussen et al. (44), hedgehogs are typically also exposed to POPs and metals that are ubiquitous in the environment. This co-exposure can cause cocktail effects that are different from those of individual compounds or even similar compounds of the same chemical group (94). In the present study, 79% of the tested animals showed co-occurrence of two or more pesticides indicating frequent co-exposure in Denmark.

Future studies on the health implications of pesticide accumulation in hedgehogs could also include biomarkers indicating e.g. liver dysfunction (bilirubin; alkaline phosphatase, ALP; alanine aminotransferase, ALT; and gamma-glutamyltransferase, GGT) in tandem with the detection of pesticides in the livers (95). There are many categories of potentially relevant biomarkers to include, such as enzymes metabolising xenobiotics as well as hematological, immunological, reproductive, endocrine, genotoxic and neuromuscular parameters, or biomarkers of oxidative stress (95).

As there are no LD_50_ doses measured in hedgehogs for the substances of our study, we have used values for rats for comparisons. The highest concentration of bromadiolone found in the hedgehogs, which was the most frequently detected compound, was 2833 ng/g, corresponding to 0.0028 mg/g. LD_50_ oral toxicity for bromadiolone is 0.4 mg/kg body weight for rats, corresponding to 0.0004 mg/g body weight (96). If the LD_50_ is the same for hedgehogs as for rats, and if the concentration of bromadiolone in the liver tissue of the hedgehog can be directly compared to the concentration per kilogram body weight given as LD_50_ for rats, the measured concentration could have been lethal for that individual. However, since other organisms typically have higher concentrations in the liver than in other organs (97), the concentration normalised to body weight will probably be lower than the liver concentration. Furthermore, the median value is 1.0 ng/g for all samples, i.e., considerably lower than the maximum concentration.

For permethrin, the LD_50_ oral dose is 480 mg/kg body weight for mammals in primary poisoning (98). This would correspond to an LD_50_ of 0.48 mg/g body weight. The highest liver concentration of permethrin in the hedgehogs was 23.8 ng/g, corresponding to 0.000024 mg/g, which is therefore presumably not a lethal concentration. It has been suggested that the LD_50_ for cats should be 100 mg/kg, corresponding to 0.1 mg/g, since cats cannot metabolise the substance (99). This may also be true for hedgehogs, but even with an LD_50_ of 0.1 mg/g, the concentrations of permethrin found in the hedgehogs would not be lethal. The relatively high occurrence of permethrin in the hedgehogs of our study (19% for *cis*-permethrin) could indicate that hedgehogs, like cats, may also have challenges in metabolising this substance, although we do not have information about the time and extent of permethrin exposure. According to a number of scientific studies, permethrin should be excreted in the urine within a few days after treatment in rats and humans (100). It could be assumed that hedgehogs could have been treated with permethrin-containing flea treatments, especially if admitted into care at a wildlife rehabilitation centre. However, most of the individuals, in which we detected permethrin, did not die in care. However, we cannot rule out the hypothetical possibility that some of the individuals found dead in the wild may have recently been treated at a wildlife rehabilitation centre.

Among the hedgehogs that contained imidacloprid, the highest concentration was 0.808 ng/g in the liver, corresponding to 8.08 × 10^−7^ mg/g. The acute toxicity of imidacloprid is low, with LD_50_ oral doses ranging from 511 to 1084 mg/kg body weight in rats varying between studies, corresponding to 0.511 mg/g body weight at the lower end of the range (101). This suggests that lethal doses of imidacloprid were not detected in the hedgehogs of our study. We found a 35% prevalence of imidacloprid in the hedgehogs examined. We are unaware of the exposure routes of this chemical in the hedgehogs included in the study, but it could be due to intentional flea treatments of the hedgehogs, or secondary poisoning caused by the ingestion of insects such as ants, which had been exposed to imidacloprid. Not all the hedgehogs included in the study carried fleas. In contrast, a study on German hedgehogs admitted into care, showed flea infestation of 91.4% of the individuals (*N* = 455/498) (43). Further studies from Germany on live hedgehogs showed a 84.6% prevalence (*N* = 64/76) (102) and a 43.7% prevalence (*N* = 93/213) (103) of fleas. In contrast, only 8% of the 74 hedgehogs examined by Gaglio et al. (104), dying in care at UK wildlife rehabilitation centres, were diagnosed with flea infestation. It is an interesting thought that hedgehogs may unintentionally become protected against ectoparasites by ingesting insects targeted with insecticides, which could explain why some hedgehogs are not infested with fleas, even though they seem very common ectoparasites of the species. An eDNA investigation of faecal samples from the hedgehogs included in the present study may provide more information on the prevalence of fleas in these samples.

### 4.5 Other sources of exposure

It is common practice that Danish hedgehogs are treated with certain pharmaceuticals and insecticides if admitted into care at a wildlife rehabilitation centre. No medicines are specifically authorised for the treatment of hedgehogs, but a practice has been created based on experience both in Denmark and in other European countries, where medicine lists and protocols for the medical treatment of hedgehogs have been developed. Another source of exposure worth considering is cat food, which garden owners frequently provide as supplementary feeding for hedgehogs. Recent studies have shown that cat food can contain pollutants, in particular POPs, that may be originating from fishmeal (105–107). Regardless of the fact that we only tested for less persistent pesticides in our study, which are therefore less likely to accumulate in fishmeal and thus cat food, cat food could be a potential a source of POP exposure to hedgehogs.

### 4.6 Conclusion

Based on this study of dead hedgehogs collected in 2016, we can conclude that hedgehogs are exposed to various pesticides. They may serve as a potential indicator species for contaminant exposure in the terrestrial environment, due to a variety of potential exposure sources, although at present, little is known about toxicokinetics or metabolisation of contaminants in hedgehogs. Given the high number of pesticides in use, further compounds would be relevant to include in future studies, for example glyphosate-based herbicides as well as the substance metaldehyde, which was the active ingredient in slug pesticides, especially applied in residential gardens in the fight against the Spanish slug (*Arion vulgaris*), until this compound was banned in Denmark in 2001. We identified few regional differences in this study and no significant influence of factors such as the hedgehogs’ age, sex, habitat type, or whether they carry endoparasites or *mecC*-MRSA on the concentrations of the compounds detected. However, the concentrations span a large range, especially for the most frequently detected rodenticides and herbicides. These peak exposures, probably occurring together with chronic exposures from omnipresent POPs, may lead to currently unexplored effects. It would be relevant to also expand the spectrum of analytes to more insecticides, as well as POPs, including BFRs, and per- and polyfluoroalkyl substances (PFAS) in future studies. Lastly, it should be prioritised to investigate the acute and long-term health effects of contaminants in hedgehogs, and the influence on the fitness of the individuals. Currently, it remains unknown, whether these pollutants pose a risk to hedgehog health as there is a lack of data on toxic threshold levels for European hedgehogs.

## Data availability statement

The original contributions presented in the study are included in the article/Supplementary material, further inquiries can be directed to the corresponding author.

## Ethics statement

Ethical approval was not required for the study involving animals in accordance with the local legislation and institutional requirements because the hedgehogs used were dead (dying from natural causes in the wild or in care at a rehabilitation centre).

## Author contributions

SR: Conceptualization, Data curation, Formal analysis, Funding acquisition, Investigation, Methodology, Project administration, Resources, Software, Validation, Visualization, Writing – original draft, Writing – review & editing. PR: Conceptualization, Funding acquisition, Investigation, Methodology, Resources, Supervision, Writing – review & editing. JN: Conceptualization, Data curation, Funding acquisition, Investigation, Methodology, Project administration, Resources, Supervision, Writing – review & editing. CP: Conceptualization, Data curation, Formal analysis, Funding acquisition, Investigation, Methodology, Project administration, Resources, Software, Supervision, Validation, Visualization, Writing – review & editing. KV: Data curation, Formal analysis, Investigation, Methodology, Project administration, Resources, Software, Supervision, Validation, Visualization, Writing – original draft, Writing – review & editing.
