# Supplementary material for: Pesticides in the population of European hedgehogs (Erinaceus europaeus) in Denmark
Source: Front Vet Sci. 2024 Aug 9;11:1436965. doi: 10.3389/fvets.2024.1436965 (PMC11341479; doi:10.3389/fvets.2024.1436965)
Supplement: Supplementary file 1 [file Data_Sheet_1.pdf]

Supplementary Material

Pesticides in the population of European hedgehogs (*Erinaceus europaeus*) in Denmark

Sophie Lund Rasmussen*, **Peter Roslev, Jeppe Lund Nielsen, Cino Pertoldi, Katrin Vorkamp**

Correspondence: Sophie Lund Rasmussen: sophielundrasmussen@gmail.com

1 Supplementary Information

1.1 Selection of rodenticides

We chose to analyse seven substances in the category of rodenticides: bromadiolone, coumatetralyl, brodifacoum, difenacoum, difethialone, α-chloralose and β-chloralose. These were selected as they were authorised for use in Denmark and could be used by everyone.

According to the Pesticide Database (1) carbon dioxide, floucomafen, aluminium phosphide as well as chlorophacinone and cholecalciferol were also authorised rodenticides in Denmark in 2016. The latter two substances are for professional use only. As cholecalciferol was not included in the list of pesticides sold in the Pesticide Statistics for 2016, and as chlorophacinone was first authorised for use in 2015, but was not reported in the statistics for 2016, we chose to exclude these from the analysis (2). The substance aluminium phosphide, which according to the Pesticide Statistics for 2016 was approved for use in 2016, was omitted from the analysis as it is a toxic gas and therefore not safe for staff to work with in the laboratory. In addition, it could not be combined with the organic compounds in the same method. We also chose to omit the rodenticide floucomafen, as according to the Pesticide Statistics for 2016, this substance was used very rarely in the years around 2016, as it was only authorised for use by professionals. It was subsequently banned for use in Denmark ultimo 2019 (1, 2).

Bromadiolone, coumatetralyl, brodifacoum, difenacoum and difethialone are all anticoagulants, which are slow-acting poisons that inhibit the blood's ability to clot. They prevent the liver from producing the substance prothrombin, which is necessary for blood to clot. Poisoned rats and mice therefore die from internal bleeding approximately one week after ingesting a lethal dose of the poison. α-chloralose (and its isomer β-chloralose) is the only non-anticoagulant rodenticide authorised for use in Denmark. The substance anaesthetises the mouse and significantly reduces metabolism, causing a lethal drop in the mouse's body temperature.

1.2 Selection of insecticides

1.2.1 Fipronil

Fipronil is a phenylpyrazole-based insecticide and acaricide (miticide) that has been commercialised since 1996 (3). It is sold, among other things, as the product Frontline, which is used as a flea repellent for dogs and cats, but also in, for example, ant bait cans (4).

Fipronil affects the neurotransmitter gamma-aminobutyrate (GABA), which controls the transmission of the electrical impulse between nerve cells. Fipronil blocks the GABA-controlled chloride channels in the central nervous system, leading to disruption of the GABA receptors, which inhibit the nerve signal by preventing the uptake of chloride ions. This disruption of the GABA receptors results in additional neurological stimulation and death in the target group, the insects (5). Fipronil binds less strongly to GABA receptors in mammals, making it less toxic to mammals than insects (6). Toxicity has not been scientifically described for hedgehogs, but a growing number of hedgehog keepers have expressed concern that fipronil may be toxic to hedgehogs, as deaths and neurological symptoms have been observed in hedgehogs treated for fleas with the product Frontline (7, 8). However, in some places it is still actively used in the care of orphaned, sick and injured hedgehogs. Fipronil was found to bioaccumulate in fish, and radioactivity originating from ^{14}C-fipronol administered to rats could also be measured in different organs e.g. livers, adrenal glands and fat tissue, with up to 25% and 75% being excreted with urine and faeces, respectively (9). Concerns have previously been expressed about the effect of fipronil on the reproduction of small insectivorous mammals that consume invertebrates poisoned with fipronil (10)as previous studies have shown that fipronil caused reduced fertility and litter size in rats (11). Fipronil can degrade to fipronil sulphone and sulphide, among other toxic substances (12, 13).

1.2.2 Imidacloprid

Imidacloprid is a neonicotinoid insecticide. Neonicotinoids act on specific receptors (nicotinic acetylcholine receptors) in the nervous system of lice and fleas, paralysing and killing them (14). To reduce toxicity in vertebrates, neonicotinoid components have been selected that are highly specific to the subtypes of nicotinic receptors found in insects. Furthermore, neonicotinoids do not cross the blood-brain barrier, which also reduces toxicity in vertebrates (15). However, the use of imidacloprid is of concern due to its effect on insects such as bees (16, 17) which has led to an EU ban in 2018 on the use of certain neonicotinoids, including imidacloprid, as pesticides in agriculture where the environment is exposed (18, 19).

Imidacloprid was among the ten most used active substances in the Danish municipalities in 2016 (20). According to the Pesticide Statistics, 2255 kg of imidacloprid was sold in Denmark in 2016 (2). Imidacloprid is used in the form of flea control products for pets in private households, as it is an ingredient in popular flea control products such as Advantage Vet, Advocate and Bayvantic. Imidacloprid-based flea control products are also used to treat fleas and other ectoparasites in rehabilitated hedgehogs.

1.2.3 Permethrin

Permethrin is a neurotoxic synthetic pyrethroid used as an insecticide. It affects the sodium channels in the membranes of nerve cells by prolonging the flow of sodium, causing depolarisation and resulting in paralysis of the respiratory organs in insects (21).

According to the Pesticide Statistics, 1778 kg permethrin was sold in Denmark in 2016 (2) for use as pesticides, including use as rodenticides and preservatives (against pests in timber). For private use, no sales of permethrin were registered in the report (22) but other pyrethroids such as pyrethrin and cypermethrin were registered. Alpha-cypermethrin is listed as one of the most commonly used pesticides on public land under municipal auspices in 2016 (20). With the high total consumption of pyrethroids, it must therefore be assumed that hedgehogs can potentially be exposed to pyrethroids in various ways in their movement in nature, and possibly also via flea treatment in care.

Permethrin is toxic to cats as they have a reduced ability to break down the substance effectively because they lack the enzyme glucuronidase, which is necessary in the transformation of permethrin through the process of glucuronidation (23). However, this is not the case for dogs. Therefore, permethrin is included in a number of flea control products for dogs. Due to its toxicity in cats, permethrin might also be toxic to hedgehogs, but this has not been scientifically confirmed.

1.3 Selection of herbicides

We selected eight herbicides for the analyses: metamitron, MCPA, 2,4-D, diflufenican, prosulfocarb, bentazone, pendimethalin and fluroxypyr. As several herbicides are authorised for use in Denmark, the selection process was based on the statistics for the consumption of the substances in the country, based on the Danish Environmental Protection Agency's reports on the consumption of herbicides among private individuals, farmers and municipalities for the year 2016. Based on the consumption described in the reports, we selected the substances among the most commonly used.

The substances MCPA, diflufenican and fluroxypyr were among the ten most commonly used active substances in municipal consumption on public land in 2016 (20). MCPA and 2,4-D were on the list of most used herbicides in private gardens in 2017 (22). All the selected substances were sold in large quantities for agricultural use in 2016, ranging from 17,292 kg (2,4-D) to 357,344 kg (prosulfocarb) (2). The list originally also included glyphosate and pelargonic acid, which were excluded after method development, as they could not be analysed with the same method.

2 Supplementary Tables

Table 1. Method detection limits (ng/g wet weight) for the selected substances in approximately 0.5 g hedgehog liver.

Compound	Method detection limit*
Coumatetralyl	0.07-0.23
Bromadiolone	0.08-0.28
Difenacoum	0.07-0.25
Brodifacoum	0.07-0.25
Difethialone	0.06-0.22
α-chloralose	0.07-0.25
β-chloralose	0.05-0.19
Imidacloprid	0.14-0.47
Diflufenican	0.07-0.23
Prosulfocarb	0.07-0.25
MCPA	0.07-0.25
2,4-D	0.08-0.26
Fluroxypyr	0.07-0.25
Metamitron	0.08-0.26
Bentazone	0.08-0.26
Pendimethalin	0.07-0.24
Fipronil	0.39-1.33
Cis-permethrin	0.05-0.18
Trans-permethrin	0.26-0.90

*Limits vary between samples due to differences in sample intake. The highest limit is related to a sample of only 0.2 g.

Table 2. Background information of the 115 hedgehogs included in the chemical analyses.

ID number	Weight of liver sample (g)	Age	Zip code	Municipality	Sex	Cause of death	Latitude	Longitude	Habitat type	MRSA Negative or Positive	Region	Endoparasites	Date of death
4	0.49	4	4800	Guldborgsund	Male	Naturally, in the wild	54.772696	11.870536	Urban area	Negative	Falster	Present	12.08.2016
14	0.58	5	8370	Favrskov	Male	Roadkill	56.364406	10.116201	Rural area		Jutland south of the Limfjord	Absent	13.06.2016
16	0.6	10	8471	Favrskov	Male	Naturally, in the wild	56.189738	10.010456	Rural area	Positive	Jutland south of the Limfjord	Present	07.07.2016
24	0.58	5	8860	Favrskov	Male	In care	56.395979	9.788539	Rural area	Positive	Jutland south of the Limfjord	Present	08.07.2016
48	0.6	4	4990	Guldborgsund	Unknown	Roadkill	54.824444	11.688611	Rural area	Positive	Lolland	Present	24.08.2016
58	0.52	5	3230	Helsingør	Male	In care	56.049957	12.379283	Rural area	Negative	Zealand	Present	29.07.2016
60	0.53	9	4990	Guldborgsund	Male	Roadkill	54.800779	11.636188	Urban area	Positive	Lolland	Absent	19.06.2016
61	0.5	4	3120	Gribskov	Male	Naturally, in the wild	56.096194	12.380117	Urban area	Positive	Zealand	Present	03.07.2016
108	0.52	1	2300	Københavns	Male	Roadkill	55.658458	12.624532	Urban area	Positive	Zealand	Present	04.07.2016
111	0.49	0	2960	Hørsholm	Female	In care	55.888799	12.534718	Urban area	Positive	Zealand	Present	25.09.2016
160	0.56	4	4200	Kalundborg	Female	Roadkill	55.428208	11.392723	Rural area	Positive	Zealand	Absent	07.07.2016
171	0.52	5	2990	Fredensborg	Male	In care	55.933359	12.500066	Urban area	Positive	Zealand	Present	22.07.2016
182	0.6	0	4800	Guldborgsund	Unknown	Roadkill	54.789865	11.943214	Rural area	Negative	Falster	Present	01.09.2016
184	0.56	0	4913	Lolland	Male	Naturally, in the wild	54.936927	11.275177	Rural area	Positive	Lolland	Present	16.10.2016
193	0.6	0	4000	Roskilde	Female	In care	55.735688	12.150509	Urban area	Positive	Zealand	Present	24.10.2016
195	0.44	0	4736	Næstved	Female	Naturally, in the wild	55.047306	12.052231	Rural area	Negative	Zealand	Absent	26.09.2016
196	0.51	0	4654	Faxe	Female	Naturally, in the wild	55.216864	12.164782	Urban area	Positive	Zealand	Absent	01.10.2016
197	0.64	1	4654	Faxe	Female	Naturally, in the wild	55.220570	12.175908	Urban area	Positive	Zealand	Present	12.09.2016
199	0.59	0	2400	Københavns	Female	Naturally, in the wild	55.694490	12.51741	Urban area	Negative	Zealand	Present	16.10.2016
202	0.59	0	2610	Københavns	Male	Naturally, in the wild	55.686061	12.458769	Urban area	Positive	Zealand	Absent	24.10.2017
206	0.54	0	2840	Lyngby-Taarbæk	Female	In care	55.819089	12.463856	Urban area	Positive	Zealand	Present	26.09.2016
207	0.54	0	2720	Københavns	Female	In care	55.697449	12.479602	Urban area	Positive	Zealand	Absent	14.10.2016
208	0.5	0	2000	Københavns	Male	Naturally, in the wild	55.690495	12.530109	Urban area	Positive	Zealand	Absent	
209	0.68	0		Fredensborg	Male	In care				Positive	Zealand	Present	21.11.2016
210	0.59	3	4780	Vordingborg	Male	Naturally, in the wild	54.987339	12.292589	Urban area	Positive	Møn	Present	30.07.2016
211	0.49	1	4771	Vordingborg	Male	Naturally, in the wild	55.009326	12.142245	Rural area	Negative	Zealand	Present	02.07.2016
212	0.59	1	4720	Vordingborg	Male	In care	55.095260	12.04732	Rural area	Positive	Zealand	Present	20.06.2016
214	0.6	1	4654	Faxe	Male	Naturally, in the wild	55.203577	12.138386	Urban area	Negative	Zealand	Present	
215	0.69	0		Vordingborg	Male	Roadkill					Zealand	Present	
220	0.54	2	3700	Bornholm	Male	Roadkill	55.116900	14.8169	Rural area	Positive	Bornholm	Present	06.12.2016
222	0.52	0	4220	Slagelse	Male	Roadkill	55.313343	11.167178	Urban area	Negative	Zealand	Present	27.09.2016
223	0.6	3	6000	Kolding	Male	Roadkill	55.496836	9.37952	Rural area	Negative	Jutland south of the Limfjord	Present	24.07.2016
225	0.54	2	6000	Kolding	Male	Roadkill	55.503043	9.4666	Urban area	Positive	Jutland south of the Limfjord	Present	27.07.2016
226	0.56	1	9210	Aalborg	Male	Roadkill	57.010304	9.944091	Urban area	Negative	Jutland south of the Limfjord	Present	Summer of 2016
227	0.5	0	9400	Aalborg	Male	Roadkill	57.065561	9.910025	Urban area	Positive	Jutland north of the Limfjord	Present	Summer of 2016
228	0.55	0	9000	Aalborg	Male	In care	57.038954	9.958226	Urban area	Negative	Jutland south of the Limfjord	Present	22.05.2016
229	0.53	4	8983	Randers	Male	Roadkill	56.571546	10.116142	Rural area	Positive	Jutland south of the Limfjord	Present	24.06.2016

230	0.64	3	7000	Fredericia	Male	In care	55.595939	9.705603	Rural area	Positive	Jutland south of the Limfjord	Present	24.06.2016
234	0.6	5	9500	Rebild	Male	Roadkill	56.633424	9.81348	Urban area	Positive	Jutland south of the Limfjord	Present	Summer of 2016
235	0.47	2	6760	Esbjerg	Male	Roadkill	55.334900	8.833487	Rural area	Positive	Jutland south of the Limfjord	Present	18.07.2016
236	0.49	1	6715	Esbjerg	Male	Roadkill	55.550278	8.471329	Rural area	Positive	Jutland south of the Limfjord	Present	26.06.2016
237	0.63	3	8600	Silkeborg	Male	In care	56.181696	9.700154	Rural area	Positive	Jutland south of the Limfjord	Present	01.07.2016
239	0.69	2	5290	Kerteminde	Female	Roadkill	55.384760	10.527314	Urban area	Positive	Funen	Absent	July 2016
240	0.54	1	5540	Kerteminde	Female	Roadkill	55.307093	10.635027	Urban area	Positive	Funen	Present	19.08.2016
242	0.52	16	8600	Silkeborg	Male	In care	56.166745	9.538739	Urban area	Negative	Jutland south of the Limfjord	Present	10.07.2016
247	0.52	0	8766	Ikast-Brande	Female	Naturally, in the wild	55.961713	9.392926	Urban area	Negative	Jutland south of the Limfjord	Present	Summer of 2016
257	0.52	1	9000	Aalborg	Female	In care	57.034865	9.919984	Urban area	Positive	Jutland south of the Limfjord	Present	22.05.2016
258	0.51	3	9000	Aalborg	Female	In care	57.038904	9.910511	Urban area	Positive	Jutland south of the Limfjord	Present	15.07.2016
262	0.65	3	8800	Viborg	Male	Roadkill	56.397222	9.726111	Rural area	Negative	Jutland south of the Limfjord	Absent	09.06.2016
264	0.61	1	8800	Viborg	Male	Roadkill	56.441944	9.409722	Urban area	Negative	Jutland south of the Limfjord	Absent	17.06.2016
267	0.5	3	4000	Roskilde	Male	Roadkill	55.612299	12.040511	Rural area	Positive	Zealand	Absent	05.08.2016
268	0.52	2	4000	Roskilde	Male	Roadkill	55.734745	12.160652	Rural area	Positive	Zealand	Present	
273	0.6	1	8464	Silkeborg	Female	Roadkill	56.151306	9.914664	Urban area	Negative	Jutland south of the Limfjord	Present	06.06.2016
278	0.5	6	8860	Favrskov	Male	Roadkill	56.434722	9.768888	Rural area		Jutland south of the Limfjord	Present	13.07.2016
281	0.58	6	9510	Rebild	Male	Roadkill	57.248956	10.006582	Rural area	Positive	Jutland south of the Limfjord	Present	29.06.2016
305	0.61	6	6640	Kolding	Male	Roadkill	55.536977	9.364187	Rural area		Jutland south of the Limfjord	Present	05.07.2016
311	0.46	2	4760	Vordingborg	Male	In care	55.043116	12.003326	Rural area		Zealand	Present	28.06.2016
312	0.58	3	3450	Allerød	Female	Naturally, in the wild	55.865790	12.327858	Urban area	Negative	Zealand	Present	28.07.2016
326	0.59	1	6715	Esbjerg	Male	Roadkill	55.498746	8.453148	Urban area	Positive	Jutland south of the Limfjord	Present	08.07.2016
327	0.52	1	2840	Lyngby-Taarbæk	Male	Naturally, in the wild	55.816592	12.511846	Urban area	Negative	Zealand	Present	29.06.2016
328	0.59	3	4250	Slagelse	Male	Roadkill	55.816592	12.511846	Urban area		Zealand	Present	31.05.2016
329	0.54	3	4200	Slagelse	Male	Roadkill	55.399248	11.32221	Urban area		Zealand	Present	19.08.2016
330	0.55	3	4700	Næstved	Male	Naturally, in the wild	55.223560	11.585252	Rural area		Zealand	Present	03.08.2016
331	0.48	3	4800	Guldborgsund	Female	Naturally, in the wild	54.756943	11.886857	Urban area		Falster	Present	21.09.2016
332	0.54	0	4800	Guldborgsund	Female	In care	54.764748	11.881477	Urban area		Falster	Present	23.09.2016
333	0.48	2	4840	Guldborgsund	Male	Naturally, in the wild	54.940548	11.832911	Rural area		Falster	Present	29.09.2016
334	0.69	0	Falster	Guldborgsund	Male	Naturally, in the wild					Falster	Present	02.10.2016
336	0.57	5	4840	Guldborgsund	Female	Roadkill	54.940548	11.832911	Rural area		Falster	Present	29.09.2016
337	0.57	0	4840	Guldborgsund	Female	Naturally, in the wild	54.902614	11.872665	Rural area		Falster	Present	10.10.2016
343	0.59	10	4242	Slagelse	Male	Roadkill	55.300332	11.259908	Rural area		Zealand	Absent	01.08.2016
345	0.7	4	4200	Slagelse	Female	Roadkill	55.403864	11.254957	Rural area		Zealand	Absent	28.07.2016
361	0.6	2	4840	Guldborgsund	Female	Roadkill	54.939157	11.752991	Rural area		Falster	Present	09.09.2016
362	0.53	0	2640	Høje-Taastrup	Female	In care	55.663633	12.200717	Urban area	Negative	Zealand	Absent	20.08.2016
365	0.58	1	3720	Bornholm	Female	Roadkill	55.084800	14.8841	Rural area	Positive	Bornholm	Present	19.10.2016
366	0.47	3	3720	Bornholm	Male	Roadkill	55.069900	14.8163	Rural area	Negative	Bornholm	Present	27.07.2016
371	0.48	0	3700	Bornholm	Male	Roadkill	55.097100	14.7358	Urban area	Negative	Bornholm	Present	06.10.2016

373	0.57	2	3700	Bornholm	Unknown	Roadkill	55.112700	14.7354	Rural area	Negative	Bornholm	Absent	07.07.2016
377	0.57	9	7000	Fredericia	Male	Naturally, in the wild	55.629155	9.557239	Rural area	Positive	Jutland south of the Limfjord	Present	06.07.2016
380	0.6	5	4160	Faxe	Female	Roadkill	55.314077	11.682319	Rural area		Zealand	Present	06.08.2016
385	0.55	4	4180	Ringsted	Male	Roadkill	55.444519	11.535777	Rural area		Zealand	Absent	26.09.2016
397	0.59	2	8464	Skanderborg	Male	Roadkill	56.156790	9.91821	Urban area	Negative	Jutland south of the Limfjord	Absent	
403	0.56	13	5250	Odense	Male	In care	55.378719	10.357522	Urban area		Funen	Present	23.10.2016
407	0.61	0	4241	Slagelse	Male	Naturally, in the wild	55.360319	11.252962	Urban area		Zealand		10.10.2016
408	0.48	0	4241	Slagelse	Male	Naturally, in the wild	55.360319	11.252962	Urban area		Zealand	Absent	10.10.2016
414	0.46	0	7160	Hedensted	Male	Roadkill	55.894740	9.525908	Rural area	Negative	Jutland south of the Limfjord	Absent	01.09.2016
428	0.59	0	7100	Vejle	Male	In care	55.734532	9.529962	Urban area	Positive	Jutland south of the Limfjord	Absent	26.09.2016
429	0.23	0	7000	Fredericia	Female	In care	55.530174	9.716283	Urban area	Positive	Jutland south of the Limfjord	Absent	29.09.2016
431	0.7	0	6000	Kolding	Female	In care	55.449907	9.421538	Rural area	Positive	Jutland south of the Limfjord	Present	03.10.2016
432	0.52	0	8722	Hedensted	Male	Naturally, in the wild	55.754026	9.719057	Rural area	Positive	Jutland south of the Limfjord	Absent	19.09.2016
433	0.2	0	9900	Frederikshavn	Female	In care	57.452804	10.517431	Urban area		Jutland north of the Limfjord	Absent	10.09.2016
434	0.64	0	9800	Hjørring	Female	In care	57.452425	10.012888	Urban area		Jutland north of the Limfjord	Present	01.08.2016
435	0.26	0	9900	Hjørring	Female	In care	57.452804	10.517431	Urban area		Jutland north of the Limfjord	Absent	10.09.2016
436	0.46	3	9900	Frederikshavn	Male	Roadkill	57.451469	10.506754	Urban area	Positive	Jutland north of the Limfjord	Present	16.07.2016
438	0.62	0	7330	Herning	Male	In care	55.952307	9.114642	Urban area	Positive	Jutland south of the Limfjord	Present	18.11.2016
439	0.57	3	8870	Randers	Female	In care	55.952307	9.114642	Urban area	Negative	Jutland south of the Limfjord	Present	30.09.2016
440	0.51	2	7860	Skive	Male	Naturally, in the wild	55.952307	9.114642	Urban area	Positive	Jutland south of the Limfjord	Present	27.09.2016
442	0.57	1	6000	Kolding	Male	Roadkill	55.378411	9.277429	Rural area	Negative	Jutland south of the Limfjord	Present	25.09.2016
445	0.63	0	5200	Odense	Female	In care	55.397066	10.344436	Urban area		Funen	Absent	26.09.2016
451	0.53	3	5800	Nyborg	Male	Roadkill	55.325067	10.807361	Urban area	Positive	Funen	Absent	11.06.2016
459	0.67	4	6600	Vejen	Female	Naturally, in the wild	55.469926	9.236335	Rural area	Negative	Jutland south of the Limfjord	Present	24.08.2016
487	0.55	1	8930	Randers	Female	Roadkill	56.482239	10.042145	Urban area	Negative	Jutland south of the Limfjord	Present	30.08.2016
489	0.55	6	6100	Haderslev	Male	Naturally, in the wild	55.218627	9.55185	Rural area	Positive	Jutland south of the Limfjord	Present	28.08.2016
490	0.59	4	8920	Randers	Female	Roadkill	56.734670	10.125432	Rural area	Negative	Jutland south of the Limfjord	Present	07.09.2016
502	0.53	4	5260	Odense	Female	In care	55.319387	10.320908	Rural area		Funen	Absent	24.08.2016
539	0.51	6	6230	Aabenraa	Female	Naturally, in the wild	55.026866	9.305407	Urban area		Jutland south of the Limfjord	Present	16.10.2011
562	0.54	5	9200	Aalborg	Female	Roadkill	57.001690	9.866017	Urban area		Jutland south of the Limfjord	Present	30.07.2016
571	0.56	11	5884	Svendborg	Male	Naturally, in the wild	55.137311	10.730383	Rural area		Funen	Present	26.06.2016
589	0.51	4	8600	Silkeborg	Female	In care	56.166612	9.553466	Urban area		Jutland south of the Limfjord	Present	01.09.2016
593	0.55	5	8380	Aarhus	Female	Naturally, in the wild	56.249876	10.150966	Rural area		Jutland south of the Limfjord	Present	29.09.2016
604	0.51	5	4200	Slagelse	Male	Roadkill	55.329357	11.398885	Rural area		Zealand	Absent	03.09.2016
631	0.54	4	5700	Svendborg	Female	Roadkill	55.036033	10.630931	Rural area		Funen		06.05.2016
671	0.5	4	6900	Ringkøbing-Skjern	Male	Naturally, in the wild	55.946342	8.481356	Urban area		Jutland south of the Limfjord		23.08.2016
684	0.52	5	8381	Aarhus	Male	Naturally, in the wild	56.188492	10.087136	Urban area		Jutland south of the Limfjord		17.08.2016
687	0.46	5	8420	Syddjurs	Female	Naturally, in the wild	56.120058	10.505771	Urban area		Jutland south of the Limfjord		13.06.2016
696	0.64	4	6400	Sønderborg	Female	Naturally, in the wild	54.915153	9.783566	Urban area		Jutland south of the Limfjord		10.10.2016

Table 3. Overview of the number of pesticides with positive detection.

**Number of the 19 compounds detected in the individual**	**Number of individuals**
0	8
1	16
2	22
3	13
4	23
5	13
6	10
7	7
8	2
9	1

Table 4. Results from the screening for the 19 selected substances in the 115 liver samples from hedgehogs, distributed across the 8 regions. Number of positive samples (N), percentage distribution of positive samples (%) i.e. samples above the method detection limit, median values of the positive samples (Median), minimum concentration above the method detection limit (Min), maximum concentration (Max). For Median, Min and Max the values are given in ng/g. The substance fipronil is omitted from the table as it was below detection limits in all samples. Note that for statistical tests, all values below method detection limits were replaced by zero, resulting in median values of 0 for the majority of compounds.

		Region	Zealand	Jutland north of the Limfjord	Møn	Lolland	Jutland south of the Limfjord	Fyn	Falster	Bornholm
		**Sample size**	37	5	1	3	47	8	9	5
**Rodenticides**										
	Coumatetralyl	**N**	16 (43.2%)	1 (20%)	1 (100%)	2 (66.7%)	17 (36.2%)	3 (37.5%)	4 (44%)	2 (40%)
		**Min.**	0.068	0.319	6.165	0.180	0.107	0.151	0.210	0.130
		**Max**	12.431	0.319	6.165	2.869	23.68	0.890	2.338	0.295
		**Median**	0.384	0.319	6.165	1.525	0.598	0.739	0.387	0.213
	Bromadiolone	**N**	28 (75.7%)	2 (40%)	1 (100%)	3 (100%)	38 (88.4%)	8 (100%)	6 (66.7%)	5 (100%)
		**Min.**	0.107	0.296	0.360	0.592	0.118	0.107	0.345	0.179
		**Max**	1189.877	1.037	0.360	272.448	2832.767	994.094	566.515	39.750
		**Median**	0.952	0.667	0.360	1.796	17.757	95.414	1.057	14.881
	Difenacoum	**N**	8 (21.6%)			2 (66.7%)	20 (46.5%)	1 (12.5%)		
		**Min.**	0.081			0.192	0.099	3.043		
		**Max**	1.412			2.409	29.718	3.043		
		**Median**	0.164			1.301	0.311	3.043		
	Brodifacoum	**N**	10 (27.0%)			2 (66.7%)	16 (37.2%)	5 (62.5%)	4 (44%)	
		**Min.**	0.096			0.103	0.097	0.118	0.189	
		**Max**	2.559			0.108	18.578	2.319	0.772	
		**Median**	0.198			0.106	0.768	0.167	0.447	
	Difethialone	**N**	14 (37.8%)			1 (33.3%)	7 (16.3%)	5 (62.5%)	3 (33.3%)	1 (20%)
		**Min.**	0.069			0.175	0.079	0.086	0.099	0.279

		**Max**	2.047			0.175	0.517	59.223	0.498	0.279
		**Median**	0.325			0.175	0.092	0.432	0.120	0.279
	α-chloralose	**N**					3 (7.0%)			1 (20%)
		**Min.**					0.088			0.233
		**Max**					0.097			0.233
		**Median**					0.093			0.233
	β-chloralose	**N**	3 (8.1%)							
		**Min.**	0.063							
		**Max**	0.086							
		**Median**	0.076							
**Insecticides**										
	Imidacloprid	**N**	17 (45.9%)	2 (40%)	1 (100%)	3 (100%)	10 (23.2%)	2 (25%)	3 (33.3%)	2 (40%)
		**Min.**	0.160	0.262	0.204	0.184	0.187	0.221	0.224	0.205
		**Max**	0.808	0.566	0.204	0.282	0.518	0.486	0.389	0.429
		**Median**	0.361	0.414	0.204	0.269	0.295	0.354	0.241	0.317
	Permethrin (cis)	**N**	8 (21.6%)	1 (20%)	1 (100%)	1 (33.3%)	6 (13.9%)	2 (25%)	2 (22.2%)	1 (20%)
		**Min.**	0.060	0.072	0.064	0.068	0.056	0.066	0.060	0.067
		**Max**	23.831	0.072	0.064	0.068	13.227	0.066	0.077	0.067
		**Variance**	70.585	0.000	0.000	0.000	28.877	0.000	0.000	0.000
		**Stand. dev**	8.401	0.000	0.000	0.000	5.374	0.000	0.012	0.000
		**Median**	0.071	0.072	0.064	0.068	0.067	0.066	0.069	0.067
	Permethrin (trans)	**N**	1 (2.7%)							
		**Min.**	0.316							
		**Max**	0.316							
		**Median**	0.316							
**Herbicides**										
	Diflufenican	**N**	3 (8.1%)		1 (100%)		7 (16.3%)	2 (25%)		

	**Min.**	0.078		0.459		0.114	0.126		
	**Max**	0.174		0.459		1.278	0.459		
	**Median**	0.114		0.459		0.248	0.293		
Prosulfocarb	**N**	1 (2.7%)			1 (33.3%)	2 (4.6%)			1 (20%)
	**Min.**	0.114			0.229	0.100			0.153
	**Max**	0.114			0.229	0.180			0.153
	**Median**	0.114			0.229	0.140			0.153
MCPA	**N**	3 (8.1%)	1 (20%)			6 (13.9%)	1 (12.5%)	2 (22.2%)	1 (20%)
	**Min.**	0.113	1.030			0.176	2.375	0.180	0.328
	**Max**	0.469	1.030			103.638	2.375	1.724	0.328
	**Median**	0.365	1.030			5.388	2.375	0.952	0.328
2.4-D	**N**	2 (5.4%)			1 (33.3%)	9 (20.9%)	1 (12.5%)		1 (20%)
	**Min.**	0.609			1.831	0.094	0.210		0.164
	**Max**	2.461			1.831	3.106	0.210		0.164
	**Median**	1.535			1.831	0.170	0.210		0.164
Fluroxypyr	**N**					1 (2.3%)			
	**Min.**					0.174			
	**Max**					0.174			
	**Sum**					0.174			
	**Median**					0.174			
Metamitron	**N**	9 (24.3%)		1 (100%)		15 (20.9%)	3 (37.5%)	3 (33.3%)	2 (40%%)
	**Min.**	0.081		7.204		0.108	0.133	0.142	0.157
	**Max**	0.481		7.204		0.729	0.959	0.397	0.162
	**Median**	0.183		7.204		0.305	0.890	0.345	0.160
Bentazone	**N**	2 (5.4%)				1 (2.3%)			
	**Min.**	0.100				0.101			
	**Max**	0.700				0.101			

	**Median**	0.400	0.101	
Pendimethalin	**N**		1 (2.3%)	1 (12.5%)
	**Min.**		0.140	0.151
	**Max**		0.140	0.151
	**Median**		0.140	0.151

Table 5. Summary of all results above method detection limits from the chemical analysis of the 115 hedgehogs included in this study (the blank fields indicate results below method detection limits).

	Rodenticides							Insecticides			Herbicides							
ID	Coumatetralyl	Bromadiolone	Difenacoum	Brodifacoum	Difethialon	α-chloralose	β-chloralose	Imidacloprid	Permethrin (cis)	Permethrin (trans)	Diflufenican	Prosulfocarb	MCPA	2,4-D	Fluroxypyr	Metamitron	Bentazon	Pendimethalin
4	0.548	1.051		0.621				0.224										
14		458.446	0.215	10.647	0.102	0.088								2.194	0.174			
16	4.141	0.954	3.723					0.518								0.231		0.14
24	0.107	0.482		0.097				0.243	13.227					0.164				
48	6.165	1.796	0.192					0.282										
58		0.459		0.178				0.408										
60	2.869	272.448	2.409	0.103				0.269	0.068					1.831				
61	1.217	3.112		0.153	0.25			0.265	0.072									
108									0.069									
111								0.565	0.073									
160	12.431	0.379						0.278								0.362	0.7	
171	0.694	1189.877	1.412	2.559	0.108													
182									0.06									
184	0.319	0.592		0.108	0.175			0.184	0.064			0.229						
193		0.114			0.173				0.06									
195		0.657			0.478		0.086	0.215			0.114	0.114	0.113	2.461				
196								0.457								0.158		
197	0.174	34.721	0.144		0.069			0.336								0.284		
199	9.456	0.107						0.361	0.061									
202	0.303	0.13		0.096	0.714			0.402								0.183		
206									23.831								0.1	
207	0.154																	
208		0.499					0.076	0.808	0.072									
209		0.163						0.16										
210		0.36						0.204			0.459					7.204		
211	0.755	3.642		1.123														
212	3.902	4.527			2.047													
214		1.165		0.23	0.293		0.063	0.403			0.078					0.481		
215		76.508	0.183		0.477													
220	0.295	39.75			0.279	0.233		0.429	0.067			0.153						
222								0.654	0.069									
223		0.118			0.118			0.225	0.059					3.106				
225		2.144						0.247	0.067								0.101	
226		2832		0.37	0.079				0.073									
227		0.296						0.262	0.072									
228		0.693						0.267	0.066			0.18		0.408				

229	0.196	103.81	0.115															
230	20.643	0.208	3.676		0.092			0.187										
234	2.144	492.959	0.099	10.269				0.323			0.569					0.305		
235		713.631	1.389	3.547							0.121							
236		0.295									1.278			0.17		0.387		
237	0.598	0.259	0.13															
239		23.881			0.432									0.21		0.89		
240		2.806		0.141	0.094				0.066									
242	1.069	78.802	0.15	0.944	0.085	0.097		0.334								0.355		
247				0.097	0.517			0.405				0.1	8.487			0.438		
257	0.58	67.912	0.18													0.127		
258		43.494		0.882														
262																0.192		
264			0.9											0.094		0.112		
267	0.206	7.777	0.133	1.271														
268	2.525	0.533	0.778	0.217				0.348						0.609		0.117		
273																		
278		1.812	0.211															
281		169.752		0.139														
305	2.227	1066	0.363	6.349														
311		0.738						0.683										
312	0.465			0.103	0.077			0.291		0.316						0.235		
326		0.587												0.167		0.195		
327		0.121						0.238										
328	0.14	38.363		0.139	0.963								0.469					
329	0.098	174.661	0.121															
330		20.786			1.198													
331	0.89	566.515		0.189	0.12													
332								0.389					1.724					
333																		
334		0.345														0.345		
336		1.062		0.272	0.498			0.241					0.18			0.397		
337	0.225	0.943																
343		0.226																
345	0.068	104.559	0.081		0.194											0.123		
361	0.21	345.294		0.772	0.099											0.142		
362		0.48																
365	2.338	15.295												0.164		0.157		
366		3.613							0.077									

371	0.13	0.179						0.205								0.162		
373		14.881											0.328					
377	0.18	1.219											1.322			0.394		
380	0.162	247.533	0.215		0.357						0.174							
385		7.072														0.081		
397		96.187											2.288					
403	0.739	994.094	3.043	2.319	1.809			0.486			0.459					0.959		0.151
407													0.365					
408																		
414													103.638					
428	0.683															0.108		
429																		
431																		
432																		
433																		
434																		
435								0.566										
436		1.037											1.030					
438		1.905																
439		0.254																
440		2.24																
442		0.261																
445		0.107																
451	0.151	166.946		0.118				0.221										
459		0.343	0.258								0.248					0.17		
487		171.991	0.369	0.416							0.354							
489		12.925	1.979	0.363				0.328								0.511		
490		74.835																
502		253.573		0.167														
539	0.116	22.588	0.156	0.238							0.114					0.404		
562	23.68	189.381		1.83									13.909	0.217				
571		2.579			0.086						0.126		2.375			0.133		
589	0.165	983.435	2.644	0.653														
593	0.182	162.507	29.718	18.578	0.081	0.093												
604		0.252																
631		305.497		0.268	58.805				0.066									
671		0.162											0.176					
684		1.29	1.775								0.202							
687	0.146	36.285	0.254											0.135		0.729		
696	4.987	874.855							0.056									

References

1. Bekæmpelsesmiddeldatabasen [Internet]. Miljøstyrelsen (2023) [cited April 2023]. Available from: https://mst.dk/kemi/database-for-bekaempelsesmidler/bmd/.
2. Miljøstyrelsen. Bekæmpelsesmiddelstatistik 2016: Behandlingshyppighed Og Pesticidbelastning Baseret På Salg Og Forbrug. Miljøstyrelsen, Danmark: (2017).
3. Beasley VR. Direct and Indirect Effects of Environmental Contaminants on Amphibians. (2020).
4. Miljøstyrelsen. Bekæmpelsesmiddeldatabasen: The Danish Nature Agency, Miljøstyrelsen (2022) [cited 2022 October]. Available from: https://mst.dk/kemi/database-for-bekaempelsesmidler/bmd/.
5. Jackson D, Cornell CB, Luukinen B, Buhl K, Stone D. Fipronil Technical Fact Sheet: National Pesticide Information Center, Oregon State University Extension Services (2009) [cited 2022 October]. Available from: http://npic.orst.edu/factsheets/archive/fiptech.html.
6. Hainzl D, Cole LM, Casida JE. Mechanisms for Selective Toxicity of Fipronil Insecticide and Its Sulfone Metabolite and Desulfinyl Photoproduct. *Chemical research in toxicology* (1998) 11(12):1529-35.
7. Stocker L. *Notes from St Tiggywinkles: The Scandal of Toxic Flea Spray*. Haddenham, UK: St Tiggywinkles Wildlife Hospital (2014).
8. Bunnell T. [Internet]. UK: Toni Bunnell (2009. [cited 2022]. Available from: https://www.tonibunnell.co.uk/hedgehog_rehab.html.
9. U.S. Environmental Protection Agency. New Pesticide Fact Sheet - Fipronil. Washington, DC: U.S. Environmental Protection Agency, Office of Prevention, Pesticides and Toxic Substances, Office of Pesticide Programs, U.S. Government Printing Office (1996) Contract No.: EPA 737-F-96-005.
10. Andrews N, Odenkirchen E, Hetrick J. Fipronil, Environmental Fate and Ecological Effects Assessment and Characterization for Section 18 Registration of in-Furrow Applications to Rutabaga and Turnips. Washington DC: Environmental Fate and Effects Division, United States Environmental Protection Agency, (2007).
11. Gibbons D, Morrissey C, Mineau P. A Review of the Direct and Indirect Effects of Neonicotinoids and Fipronil on Vertebrate Wildlife. *Environmental Science and Pollution Research* (2015) 22(1):103-18. doi: 10.1007/s11356-014-3180-5.
12. Singh NS, Sharma R, Singh SK, Singh DK. A Comprehensive Review of Environmental Fate and Degradation of Fipronil and Its Toxic Metabolites. *Environmental Research* (2021) 199:111316. doi: https://doi.org/10.1016/j.envres.2021.111316.
13. Cravedi JP, Delous G, Zalko D, Viguié C, Debrauwer L. Disposition of Fipronil in Rats. *Chemosphere* (2013) 93(10):2276-83. doi: https://doi.org/10.1016/j.chemosphere.2013.07.083.
14. Ensley S. Chapter 44 - Imidacloprid. In: Gupta RC, editor. *Veterinary Toxicology*. Oxford: Academic Press (2007). p. 505-7.
15. Yamamoto I, Yabuta G, Tomizawa M, Saito T, Miyamoto T, Kagabu S. Molecular Mechanism for Selective Toxicity of Nicotinoids and Neonicotinoids. *Journal of Pesticide Science* (1995) 20(1):33-40.
16. Rundlöf M, Andersson GK, Bommarco R, Fries I, Hederström V, Herbertsson L, et al. Seed Coating with a Neonicotinoid Insecticide Negatively Affects Wild Bees. *Nature* (2015) 521(7550):77-80.

17. Authority EFS. Peer Review of the Pesticide Risk Assessment for Bees for the Active Substance Imidacloprid Considering the Uses as Seed Treatments and Granules. *EFSA Journal* (2018) 16(2):e05178.
18. EU. Kommissionens Gennemførelsesforordning (Eu) 2021/621 Af 15. April 2021 Strasbourg: EU, (2021).
19. EU. Commission Implementing Regulation (Eu) 2018/783. Strasbourg: (2018).
20. Miljøstyrelsen. Undersøgelse Af Forbruget Af Pesticider På Offentlige Arealer I 2016. Miljøstyrelsen, Danmark: (2018).
21. Imamura L, Hasegawa H, Kurashina K, Hamanishi A, Tabuchi A, Tsuda M. Repression of Activity-Dependent C-Fos and Brain-Derived Neurotrophic Factor Mrna Expression by Pyrethroid Insecticides Accompanying a Decrease in Ca2+ Influx into Neurons. *Journal of Pharmacology and Experimental Therapeutics* (2000) 295(3):1175-82.
22. Miljøstyrelsen. Salg Af Pesticider Til Brug I Private Haver 2017. Miljøstyrelsen, Danmark: (2019).
23. Dymond NL, Swift IM. Permethrin Toxicity in Cats: A Retrospective Study of 20 Cases. *Australian Veterinary Journal* (2008) 86(6):219-23. doi: https://doi.org/10.1111/j.1751-0813.2008.00298.x.
